# Growing pains in local food systems: a longitudinal social network analysis on local food marketing in Baltimore County, Maryland and Chester County, Pennsylvania

**DOI:** 10.1007/s10460-021-10199-w

**Published:** 2021-02-22

**Authors:** Catherine Brinkley, Gwyneth M. Manser, Sasha Pesci

**Affiliations:** Department of Human Ecology, University of California, 1 Shields Avenue, Davis, CA 95616 USA

**Keywords:** Social network analysis, Local food systems, Peri-urban, Alternative food networks, System of food systems, Complex adaptive systems

## Abstract

Local food systems are growing, and little is known about how the constellation of farms and markets change over time. We trace the evolution of two local food systems (Baltimore County, Maryland and Chester County, Pennsylvania) over six years, including a dataset of over 2690 market connections (edges) between 1520 locations (nodes). Longitudinal social network analysis reveals how the architecture, actor network centrality, magnitude, and spatiality of these supply chains shifted during the 2012–2018 time period. Our findings demonstrate that, despite growth in the number of farmers’ markets, grocery stores, farms and restaurants in both counties, each local food system also experienced high turnover rates. Over 80% of the market connections changed during the study period. Farms, farmers’ markets, and grocery stores showed a 40–50% ‘survival’ rate, indicating their role in sustaining local food systems over longer time periods. Other actors, such as restaurants, had a much higher turnover rate within the network. Both food systems became more close-knit and consolidated as the center of gravity for both local food systems pulled away from urban areas toward rural farmland. Evidence of both growth and decay within local food systems provides a new understanding of the social networks behind local food markets.

## Introduction

This research maps the evolution of two local food systems over time in order to understand broader trends in the evolution of local food marketing. The trajectory and pace of change within local food networks offers clues about how rapidly the parts of the sum evolve. Local food systems are thought to strengthen social ties between growers and eaters (Hinrichs 2000), giving a sense of community and shared social values that translate into shared political agendas (Obach and Tobin 2014). The resulting “alternative food network” (AFN) connects and mobilizes people toward “civic agriculture” (Lyson and Guptill 2004; Lyson 2012) forming what some scholars consider to be a social movement (Huey 2005; Starr 2010; Levkoe 2014) that, at times and in certain communities, advocates for farmland preservation (Brinkley 2018) and/or food justice (Allen 2008, 2010; Alkon and Norgaard 2009; Alkon and Agyeman 2011; Sbicca 2012). Local food activists tout broad promises of transformation, from improving diets that promote individual health (McNamee 2007; Waters 2011; Slocum 2011; Prosperi et al. 2019) to landscape-level changes (Vaarst et al. 2018) that reduce urban sprawl (Lima et al. 2000; Wekerle and Jackson 2005), boost local economies (Brown and Miller 2008; Winfree and Watson 2017; O’Hara and Shideler 2018), and enhance ecological sustainability (DeLind 2002; Horrigan et al. 2002; Altieri 2018). Investment in these promises occurs through purchasing food labeled as “local” and supporting markets that carry and advertise such food (Howard and Allen 2006, 2010; Eden 2011). In sum, local food systems engage people in more than just social connectedness–they also prompt collective action against the status quo by reorienting markets (McAdam 2003).

The notion of “local food” is not a monolith, nor is there a neat dichotomy between “global” and “local” (Hinrichs 2003). The boundaries of what constitutes “local” are blurred; the benefits of local food networks vary by community; and priorities and allegiances shift over time. In interviewing Community Supported Agriculture subscribers, Schnell (2013) finds that the notion of “local” is not an objective spatial denotation, but a social contract between food producers and consumers who share similar values. Local food may be considered food grown and consumed within 100 miles (Smith and MacKinnon 2007) or 100 yards (Schnell 2013). Food that is advertised as “local” is not always produced with the same values. While some farming operations may emphasize fair labor, not all do (Born and Purcell 2006). Further, many farmers change their positions over time on a variety of issues, from organic agriculture to animal welfare certifications. As such, this research explores the heterogeneity and changes in social ties across a variety of local food distribution practices without imposing limitations on distance.

## Analytical framework: understanding network architecture

Social Network Analysis (SNA) can help food scholars understand the future trajectory of local food systems, and can help reveal locations where marketing networks are realigning with concurrent social movements. SNA is used to examine ties/relationships between network actors, such as individuals or, in our research, individual markets and farms. SNA statistics help elucidate which actors are central, and presumably more influential, to a network, playing a coordinating or broker role in transmitting knowledge, values, and political agendas. In addition, SNA can quantify the architecture of groups within a network and highlight where there are rifts or mutually reinforcing relationships. SNA has been used to understand social movements where the constellation of actors and organizations involved influences the outcomes (Andrews 2001; Andrews and Edwards 2004) changing how rapidly a movement can build alliances (Knoke 1990), share ideas and practices (Gerlach 1971), coordinate activities (Staggenborg 1998), legitimize political organization (Hadenius 2001), and prompt change (Andrews 2001; Andrews and Gaby 2015; Biggs and Andrews 2015).

SNA can help scholars predict if local food systems are stable, growing, or shrinking. There is a common narrative among scholars and policy-makers that local food systems have been steadily growing (Low et al. 2015; Martinez et al. 2010). Acknowledging the rise of local food systems, the United States Department of Agriculture (USDA) began collecting direct marketing data for the agricultural census in 2002, finding a 32% increase in the percentage of direct-market sales from 2002 to 2007, and a 5.5% increase in the number of farms with DTC sales between 2007 and 2012 (Low et al. 2015). In 2012 nearly 8% of farms in the United States marketed foods locally, which the USDA defines as either direct-to-consumer (DTC) sales, such as farm stands, You-Pick operations, farmers’ markets, or Community Supported Agriculture (CSA), or sales through intermediaries such as restaurants, grocery stores, schools, hospitals, or other businesses (Low et al. 2015; Martinez et al. 2010). Intermediated markets account for two-thirds of local food sales (USDA NASS 2017) and are slowly gaining more research attention (Dimitri et al. 2019). In addition, short supply chains can connect farmers to consumers through food donations or urban gardening, where food is shared but not sold (Vitiello et al. 2015). These relationships are not tracked by the agricultural census, but may be just as important to civic agriculture (Lyson 2012).

On the other hand, some argue that local food networks are transient. Small scale farms make up the majority of those participating in local food systems (Kirschenmann et al. 2008) with 85% of farms that sell in local markets earning less than $75,000 in gross cash income in 2012 (Low et al. 2015). These smaller-scale operations spend considerable time and effort in marketing, while also being under constant threat as they compete for marketing contracts against larger growers. Additionally, some researchers have emphasized the perils of farming on the edge of urban development (Hart 1990; Kirschenmann et al. 2008). Landowners located on the periphery of growing urban areas are often tempted to sell farmland for more lucrative housing development (Kirschenmann et al. 2008). As urban areas grow outward, land values rise, creating a peri-metropolitan “bow wave” of higher prices that also increases the cost of doing business by raising land values and taxes for farmers (Hart 1991; Martellozzo et al. 2015). Indeed, increased suburbanization has resulted in loss of prime agricultural land (Seto and Ramankutty 2016). For this reason, local food proponents often tie local food systems to attempts to rescue farmland from the avalanche of urban development. For example, non-profit farmland preservation groups spend up to $124,000 per acre to buy development rights and preserve land in agriculture (Brinkley 2012). Although many customers are willing to pay nearly double the price for locally-grown food products (Brown and Miller 2008; Darby et al. 2008; Feldmann and Hamm 2015), these trends do not necessarily translate into stable local food networks. As shown by an autopsy on 32 farmers’ market closures in Oregon, even as new local food outlets arise, many fail within a few years of opening, in part due to “individualized, complex issues that are internal and/or external to the market” (Stephenson et al. 2008).

Although the agricultural census measures the total number of participating farms and the composition of marketing methods, little is known about how individual farms and markets connect to one another, and how those marketing connections change over time. Some scholars posit that the increased trust and personal relationships characteristic of local food systems creates enduring social ties (Starr et al. 2003; Chesbrough et al. 2014) based on “bonding” social capital (Putnam 2000) that would lead to long-term relationships and stable growth. In support, relationships that form through supply chain networks of local food systems exhibit transparency, a hallmark of trust (Hinrichs 2000, 2003). For instance, restaurants often promote their local suppliers as part of their routine advertising efforts, and diners build loyalty with the farms that grew the products they consume (Starr et al. 2003; Chesbrough et al. 2014; Brinkley 2017, 2018). This interpretation of local food systems would lead researchers to assume that local food system growth reported in the agricultural census is a result of the addition of new members to a stable and growing cohort. On the other hand, cumulative pressures on local food systems would indicate that while there may be overall local food system growth, actors and market channels may shift or die off at high rates, particularly at the urban edge. In such cases, the local food system would be made up of what Granovetter refers to as “weak ties” (Granovetter 1977, 1983), defined as loose affiliations that can nimbly innovate. Arguably, communities with “bridging” social capital (weak ties across groups) as well as “bonding” social capital (“strong ties” within groups) may be the most effective in organizing for collective action (Granovetter 1973; Putnam 2000). SNA can be used to visualize and quantify the spatiality and social clustering of relationships in the local food system as it changes over time, helping to make sense of underlying drivers and limits to local food system change and its affiliated social impacts.

Broadly speaking, alternative food movements have been shifting priorities and increasingly incorporating concerns for food justice (Pothukuchi and Kaufman 1999; Hammer 2004; Wekerle 2004; Horst et al. 2017), but little is known about how these shifts prompt changes in the architecture of their constituent market networks. As activists conceptualize scaling up the political ambitions of alternative food movements (Blay-Palmer et al. 2016), SNA of network architecture and change over time can illustrate how to move toward a globally interlinked network of local food systems. Such changes may be complex, as social values differ across marketing pathways and from community-to-community, and they also shift over time. The longitudinal, comparative research that we present here offers a starting point for understanding where a network of local food systems builds into larger scale social movements. For example, Hinrichs (2000) theorized that CSA members have more rural-focused values (e.g., concerns for soil health and ecological sustainability) than consumers who shop at urban farmers’ markets, thus shaping the social relationships formed within these market pathways. One might expect communities with more prominent CSA presences to have a greater focus on farmland protection and growing practices. In addition, local food systems have internal feedback loops; for example, O’Hara and Shideler (2018) found that increasing DTC food sales prompted increased sales at restaurants in metropolitan counties. Thus, a better understanding of the heterogeneity in market channels offers insights into which locally-oriented markets may grow in the future and how their growth may shift their political attention.

To build toward the above, this research uses SNA to understand how local food system networks evolve. Scholars have only recently started to apply SNA to the study of food systems. Lucy Jarosz (2000) called for the combined use of network theory and supply chain analysis for regional food systems. Two decades later, Trivette (2019) utilized SNA on 687 farms and 702 retailers across a three-state region in New England to reveal the central role of grocery stores and restaurants in local food systems. In addition, Brinkley (2017, 2018) applied geo-social network analysis to understand the extent to which local food systems are socially and geographically embedded in the two study counties used in this research, finding evidence of the local food system’s impact on land-use policies. Our research contributes to these pioneering methodological efforts and is the largest SNA of local food systems in scale, and the first to utilize longitudinal data to examine change over time.

## Methods

### Case selection

This study focuses on the local food systems of Chester County, Pennsylvania and Baltimore County, Maryland, both of which are located in peri-urban areas of the northeastern United States, in close proximity to the large urban markets of Philadelphia, Baltimore, New York City, and Washington D.C. These counties have a long history of direct marketing and local food distribution channels (Brinkley 2017). The 2012 food network data was previously collected in both counties (Brinkley 2017, 2018), thus allowing for a novel, longitudinal approach to food systems network analysis. This research compares data collected in 2012, and again in 2018. Both counties show flux within their agricultural sectors, which make them interesting cases for comparison. Baltimore County saw an 8% increase in acreage of farmland within the county from 2012 to 2017 (USDA National Agricultural Statistics Service 2017). However, although the number of farmers’ markets increased by 40% (12 to 17) from 2009 to 2016, from 2007 to 2012 Baltimore County also saw a 30% decrease (128 to 91 farms) in the number of farms that sell through direct marketing (USDA Food Environment Atlas). From 2012 to 2017, Chester County saw an 8% reduction in acreage of farmland within the county (USDA National Agricultural Statistics Service 2017). Chester County also saw a 7% increase (194 to 208 farms) in the number of farms that use direct-market channels from 2007 to 2012, and a 260% increase (3 to 11) in the number of farmers’ markets 2009–2016 (USDA Food Environment Atlas).

### Data collection

Social networks are comprised of “nodes,” which are the actors or members of the network, and “edges,” which are the ties or relations linking the nodes. Data collection was limited to raw agricultural products, rather than processed food or inedible value-added products (Table 1). Nodes include the farm, as well as the location of its first point of sale or donations (Table 2). The basis of ties (edges) between actors is the distribution of food, both via sales and donations. Based on the USDA definition of local food, sales could be made directly to consumers via CSAs, farmers’ markets, and you-pick operations, or to intermediaries, such as restaurants, distributors, grocery stores, food banks and institutions (Table 3).

**Table 1:** Node and edge table summary statistics 2012 and 2018

	Chester County	Baltimore County	Chester County	Baltimore County
	Nodes		Edges	
Only 2012	393	186	738	539
Only 2018	360	284	684	495
Both 2012 and 2018	210	116	162	116
Total	963	568	1584	1108
Confirmed closed	19	36	30	125

Confirmed closures amongst edges represent the subsequent loss of connections as a result of node closures

**Table 2:** Node table summary statistics by type of outlet for Baltimore County and Chester County, including actors that were present only in 2012, actors that were present only in 2018, and actors that were present in both 2012 and 2018

Nodes by type	2012 only	2018 only	Both 2012 and 2018	Survival rate of 2012 nodes (%)	Network growth 2012–2018 (%)	
Baltimore County, Maryland						
Farm	56	66	37	40	11	
Farmers’ market	16	20	14	47	13	
Grocer	23	28	20	47	12	
Restaurant	35	84	30	46	75	
Other	56	86	15	21	42	
Chester County, Pennsylvania						
Farm	81	122	91	53	24	
Farmers’ market	15	23	18	55	24	
Grocer	22	43	18	45	53	
Restaurant	45	57	15	25	20	
Other	230	115	68	23	− 40	

Additionally, the table includes the survival rate of 2012 nodes, calculated as [both 2012 and 2018]/([both 2012 and 2018] + [2012 only])

**Table 3:** Summary network analysis statistics 2012 and 2018

	Baltimore County		Chester County	
	2012	2018	2012	2018
Average degree	2.023	1.377	1.44	1.442
Network diameter	6	3	8	5
Graph density	0.007	0.003	0.002	0.0003
Average clustering coefficient	0.035	0.023	0.023	0.023
Average path length	1.919	1.139	2.762	1.976

We focused on nodes and edges that are transparent, meaning that connections are publicly documented. Data were collected through the review of publicly available online information, including *LocalHarvest.com,* county documents, and the official websites and social media pages (including Facebook and Instagram) of farms, restaurants, farmers’ markets, food banks, food pantries, and schools. Snowball sampling was then used to identify other actors and their relationships in the network. For example, the first node added to the 2018 Baltimore County dataset was a farmers’ market. The farmers’ market website listed all the vendors that sell at the market, thus enabling us to capture the second node in the dataset: a farm also located within the county. From node two’s website, we were able to capture their extensive list of direct sales relationships, which included actors both inside and outside of the county. We also logged attribute information for each node, including the name of the business, business address (recorded as latitude and longitude), identification number, an agricultural production typology code (Tables 4, 5), website address, contact information, and notes on how the node was found. Edges were coded based on the types of relationship they represented (e.g. wholesale, CSA, farm stand, donations). For instance, a relationship between a farm and a farmers’ market was coded as “farmers’ market” in the edge table. Table 6 in the Appendix shows the coding guide and relationship typologies captured.

The boundary that we set for this study was spatially defined by the political delimitation of each county (Chester County, Pennsylvania and Baltimore County, Maryland). We only captured relationships that involved at least one actor located within the county. As a result, we also included farms outside of Chester and Baltimore Counties that distribute their product into the county (for instance, if a farm from another county sells raw products at a farmers’ market within the county). Similarly, we also captured relationships between farms located within one of the study counties, and sales outlets located outside of their respective county. However, we only captured instances in which the products would be distributed via ground transportation.

### Data preparation

For both counties the data from 2012 to 2018 were merged into a single dataset using an R script. Edges and nodes were then individually coded based on whether they were unique to the 2012 data set, unique to the 2018 data set, or present in both data sets. In 2018, we cross checked the nodes in each dataset to find establishments that appeared to have closed since 2012. Closures were denoted in our datasets.

### Social network analysis and visualization

The SNA software package Gephi was used to visualize the network graph and run descriptive statistics on the network data. The network was visualized using the force-directed *Fruchterman Reingold* projection, which places nodes connected by an edge in relatively close proximity with one another (Fruchterman and Reingold 1991). The force-directed, multilevel *YuFan Hu* projection was also used. This projection uses coarsening and clustering to simplify the output graph (Hu 2005). Finally, we also used Gephi’s *GeoLayout* plugin, which allows for the integration of geospatial analytics, in order to visualize the spatiality of the network. Visualization in the exploratory stage of the analysis allowed us to identify apparent hubs in the network, which are nodes that have high in-degree (incoming) or out-degree (outgoing) connections across the network. We identified intermediaries and hubs by running statistics on degrees of centrality and clustering coefficients. We performed descriptive statistics for changes in the numbers of nodes and edges between 2012 and 2018, as well as changes in the distribution of types of sales outlets. Using online business profiles on *Yelp.com*, *Google*, business websites, and social media we manually calculated the percentage of establishments that appear to have closed since 2012, and we used Gephi to calculate the proportion of connections that have been lost due to these business closures.

### Limitations

Because data was manually scraped from the web, the network data is limited by how up-to-date and extensive the various actors’ publicly available information is. This is also a challenge faced by previous studies that have applied SNA to local food systems (Trivette 2019; Brinkley 2017, 2018). Although there is an economic incentive to keep distribution channels up-to-date for all of the actors involved, we know that not all of this data is an accurate reflection of the network. For example, many producers still listed restaurants that had recently closed on their list of distribution partners. Second, data on closures in the network are likely incomplete. Business profiles on *Yelp.com* and *Google* report which restaurants and grocery stores have closed, likely because those types of locations are often visited by the general public. However, because not all farms, farmers’ markets, and small vendors maintain a robust public-facing web presence, it is often difficult to tell if they are still in operation. Third, in addition to utilizing manual web scraping, the 2012 datasets were supplemented with online surveys (Brinkley 2017, 2018), which accounted for 195 nodes and 210 edges, with 90% of these in the “Other” category for node type (Table 2). Surveys were not used to augment the 2018 data set. Arguably, therefore, the 2012 dataset includes more comprehensive information on the local food network. As a result, comparisons of the 2012 and 2018 datasets become less accurate, particularly in terms of magnitude. At the same time, however, smartphone ownership has skyrocketed from 35% in 2011 to 81% in 2019 (Pew 2019), and the prevalence of online marketing has likely increased in tandem, thus arguably making online marketing a more robust data source in 2018 when compared to 2012. Last, the data provided in this research omits numerous actors in the local food system, most notably consumers. Consumers play a large role in driving and (re)orienting the food system, local and otherwise.

## Results

SNA is a powerful tool in quantitative analysis. Social networks are comprised of nodes—which are the actors, or members, of the network—and edges—which are the ties, or relations, linking the nodes in the network. Nodes may have one or more relation, and types of relations, with each other (Marin and Wellman 2011). For example, a farm might sell produce to consumers at a farmers’ market. However, the same farm might *also* utilize their booth at the same farmers’ market as a CSA pickup site. As such, there would be two edge connections between the farmers’ market and the farm: one denoting DTC sales via farmers’ market sales unrelated to the CSA, and another denoting DTC sales through a CSA-based relationship. This distinction is important because, as Hinrichs (2000) notes, CSAs and farmers’ markets offer differently embedded social relationships. Although farmers’ markets enable face-to-face interactions between farmers and consumers, they are not necessarily developing longer-term continuous relationships (Hinrichs 2000). On the other hand, the CSA model can foster greater trust and value-driven relationships between customers, who buy shares for the growing season, and CSA farmers, who are commonly motivated by non-economic factors and set share prices that are not exclusively profit-driven (Galt 2013). Such relationships may have different staying power over time, or allow for different evolutions across the network as farms transition from one form of marketing to another. We are able to explore both relationships over time using SNA.

### Growth and death

To start, we provide a descriptive comparison of both counties and the proportion of network actors and ties, then we explore change over time and network architecture. Although Chester County has a larger local food system network, both in terms of nodes and edges, the overall local food network of Chester County is shrinking, while the local food network of Baltimore County is growing (Table 1). During the 6-year study period, Baltimore County saw the addition of 284 new nodes and 495 new edges in the network. During the same time period, Chester County saw the addition of 360 new nodes, and 684 new edges, but lost 393 nodes and 738 edges (Table 1). One possible explanation is that local food systems may reach a point beyond which added growth is very difficult, due to plateauing consumer interest (Low et al. 2015) or market saturation. However, when delineated by category (Table 2), all sectors within the Chester County local food system are growing. The one exception is the “Other” category which is primarily comprised of sales and donations to institutions and civic organizations. This category relied more heavily on 2012 survey data to uncover the many farm-to-food bank donations across Chester County. Such donations are not as readily advertised on farm websites and may therefore lead to under-counting in the 2018 dataset. This finding points to nuances in how local food system growth is tabulated both in research, such as this, and by the agricultural census, where categories are broad and may overlook central connections like that of the Chester County Food Bank.

Both networks show substantial change from 2012 to 2018, with a relatively high rate of turnover of actors within the network (Table 2). When examined by node or edge category, both counties show nearly equal rates of growth *and* death in network actors (nodes) and their marketing relationships (edges). Despite growth in many categories, more than half of the participants in the local food system changed over the 6-year period, with only 40% of Baltimore County’s 2012 nodes found in the 2018 data, and only 35% of Chester County’s 2012 nodes found in the 2018 data. More telling, the connections across the network changed even more than the actors themselves, with only 18% of edges staying the same across both 2012 and 2018 in both counties. The fluctuation in edges indicates that, while actors may be stable, their relationships with one another evolve.

The rates of endurance by category varied. In the Chester County dataset, the following nodes endured: 91 farms, 23 schools involved in farm-to-school and food bank connections, 18 farmers’ markets, 18 grocery stores, 15 restaurants, 11 churches involved in food bank gardening and distribution, and 3 food banks. These locations accounted for 85% of the actors that endured from 2012 to 2018. The rest of the actors were CSA drop-off locations, community gardens, and food hubs. By comparison, the Baltimore County dataset showed 37 farms, 30 restaurants, 20 grocery stores, and 14 farmers’ markets active in the network in both 2012 and 2018. These actors made up 87% of the actors that endured within the dataset. The remaining enduring actors include CSA drop-off locations, two schools, two catering companies, and two churches.

Generalizations across categories are shown in Table 2. In 2012, the Chester County “Other” node category included 80 civic organizations (e.g., schools, churches, and retirement communities), many with gardens that donated food to other civic organizations. These gardens largely catered to schools or the Chester County Food Bank. The Chester 2012 data in the “Other” category also included 88 CSA drop-off locations. While the number of restaurants, farmers’ markets, farms, and grocers increased over the 6-year period, the miscellaneous category decreased, with a decrease in both civic organizations and CSA drop-off locations (Tables 1 and 2). This change is likely because the number of gardens associated with the food bank and other civic organizations were not as readily found online in 2018. Similarly, the 2018 Baltimore County “Other” category included 15 churches and 3 food banks.

Importantly, the “Other” category is larger than any other category across both counties. This indicates the variety of actors beyond farms, farmers’ markets, restaurants and grocers, which are currently the main focus of much of local food systems research. The “Other” category also captures new marketing typologies that may tap into other socio-political movements. For example, the 2018 Baltimore County dataset included a recently legalized cannabis shop, which purchases infused honey from a local beekeeper. Although the cannabis shop typology was collapsed into the “Other” category for our analysis, this represents a new aspect to local food systems that warrants further investigation, particularly as hemp-derivatives become more common in other local food spaces, such as farmers’ markets, and as local food systems spread into new spaces with their own divergent or intersectional political objectives.

Separating network actors into categories allows us to explore further properties of local food system stability. For example, farmers’ markets were the most stable nodes within the network across both counties. This may be because farmers’ markets generally have an explicit goal of providing business opportunities for local food producers, thus making them a relatively stable outlet for local food system sales. More than half (55%) of the farmers’ markets stayed open in Chester County through the 6-year study period, and nearly half of them (47%) stayed open in Baltimore County. This finding supports USDA agricultural census information, noting that in 7 years (2009–2016), the number of farmers’ markets increased by 270% (3 to 11) in Chester County and by 40% (12 to 17) in Baltimore County (USDA Food Environment Atlas). However, our data also show high rates of turnover, with over 40% of the 2012 farmers’ markets no longer in operation by 2018. This flux over the course of a 6-year period indicates a certain degree of market instability, as well as rapid evolution in how consumers interact within an ever-changing local food system.

Across both counties, grocers also appeared to be relatively stable actors in the local food system, with a little less than half (45% and 47% in each county) of the 2012 grocers remaining in the 2018 local food network (Table 2). Because grocers are important intermediaries that are often central to local food networks (Trivette 2019; Brinkley 2017, 2018), their relative stability in the network offers promise for long-term stability and growth in local food systems. The two counties in this study differ in terms of the growth of this food system actor, with grocers making up the largest growth (53%) in the actor category for Chester County, but not Baltimore County (12%) (Table 2). Baltimore County’s local food system is comparatively more reliant on restaurants. This might explain the greater growth in the restaurant category, with the addition of 84 new restaurants between 2012 and 2018. Although 30restaurants remained in the Baltimore County local food network throughout the course of the study, a nearly equal number of restaurants (35) also dropped out of the network between 2012 and 2018 (Table 2). The restaurant category had higher turnover in both counties when compared to grocers.

Our data indicate that, unlike restaurants, farms have greater staying power. They are also increasingly joining the local food system in both study counties. Although the USDA agricultural census noted a 30% decrease in the number of farms (128 to 91 farms) that sell through direct-market channels from 2007 to 2012 in Baltimore County (USDA, Food Environment Atlas nd), our data shows an 11% increase in the number of farms in the local food system (Table 2). Similarly, the USDA agricultural census notes a modest 4% increase in farms that sell through direct-market channels in Chester County (from 735 to 782) throughout 2007–2012; our research indicates that this county saw a 25% increase in the number of farms involved in the local food system (Table 2). The differences in figures could be because our data also capture farms that sell through intermediate markets. Intermediate markets account for two-thirds of local sales (USDA NASS 2017). Further, the offset in years between the USDA agricultural census data collection and this study may also explain the difference in figures. Also of note, the Baltimore dataset appears to capture a more representative sample of direct-market farms compared to the census, while the Chester County dataset captures about 30% of direct-market farms compared to the USDA agricultural census. This may partly be because Chester County has a large portion of Amish farms that may take part in the agricultural census, but may not have an online presence as a result of religious restrictions on technology use. Due to the nature of online data collection methodology employed in this study, we were not able to verify these Amish farms and, as a result, we could not access their marketing connections.

Confirmed business closures between 2012 and 2018 provide supporting evidence for the broad categorical trends above. Importantly, closure is distinct from actors simply dropping out of the network, as closure implies a complete and indefinite severing of network ties. Uniquely, SNA allows us to assess the disproportionate impact that the loss of specific actors can have on a network. Restaurants made up 60% of the 36 confirmed closures in Baltimore County. The second highest category of closures were farms, which represented an additional 16% of total closures. Similarly, half of the Chester network’s nineteen confirmed closures were restaurants (Table 1). Additionally, four grocery stores, three farms, two farmers’ markets, and one CSA distribution location closed, thus removing them from the 2018 network.

If a local food system is more dependent on restaurants, the flux within the network could be greater, as is the case in Baltimore County. The Baltimore County dataset shows a greater loss of nodes in terms of confirmed closures, with 12% of the nodes from 2012 having closed by 2018. This resulted in a 20% loss of edge connections, as compared to a 3% loss rate for nodes and edge connections in the Chester County dataset. Restaurants have a median lifespan of 4.5 years (Luo and Stark 2014), and other network actors may have a longer business lifespan, thus translating to increased stability within the network. Many restaurants that close see the owners or chefs establish new eateries shortly thereafter. Future research could track such transitions to see if relationships are re-established with the same farmers and distributors as new spaces open up, or if restaurants that source locally have different survival rates than their non-locally sourcing counterparts. Another possible explanation is that local food systems may need to achieve critical mass in order to compete with larger-scale food supply chains. It is possible that Chester County’s large local food system has less flux compared to the still growing local food system of Baltimore County.

Another way to view the confirmed closures is that each actor is a unique contributor to the local food system. The confirmed closure of 36 actors in the Baltimore County network had a disproportionate impact on edge connections, resulting in 125 lost relationships. Conversely, while Chester County also saw the closure of a few actors (19), those closures only resulted in the loss of 30 edge connections. In Baltimore County, the closure of five actors, in particular, resulted in a substantial loss of edges. These actors included the following restaurants and farms: Simmer Rock Farm, Atwater’s Ploughboy Kitchen, Big City Farm, Woodhall Wine Cellars, and Clementine Restaurant. Simmer Rock Farm opened in 2010 and closed by 2013, resulting in the loss of 25 connections, including three farmers’ market sales locations, 15 restaurants that carried their food, one grocery store, and a CSA. The restaurant Atwater’s Ploughboy Kitchen also closed, resulting in the loss of 37connections. Big City Farm was a collection of urban farmers; its closure resulted in the loss of 14 connections, and the closure of Woodhall Wine Cellars and Clementine restaurant both resulted in the loss of seven connections. Collectively, these account for the 72% of lost connections due to closures within the network, pointing to the significant impact that a few actors can have on local food system dynamics.

### Visualization of network architecture

**Fig. 1:**
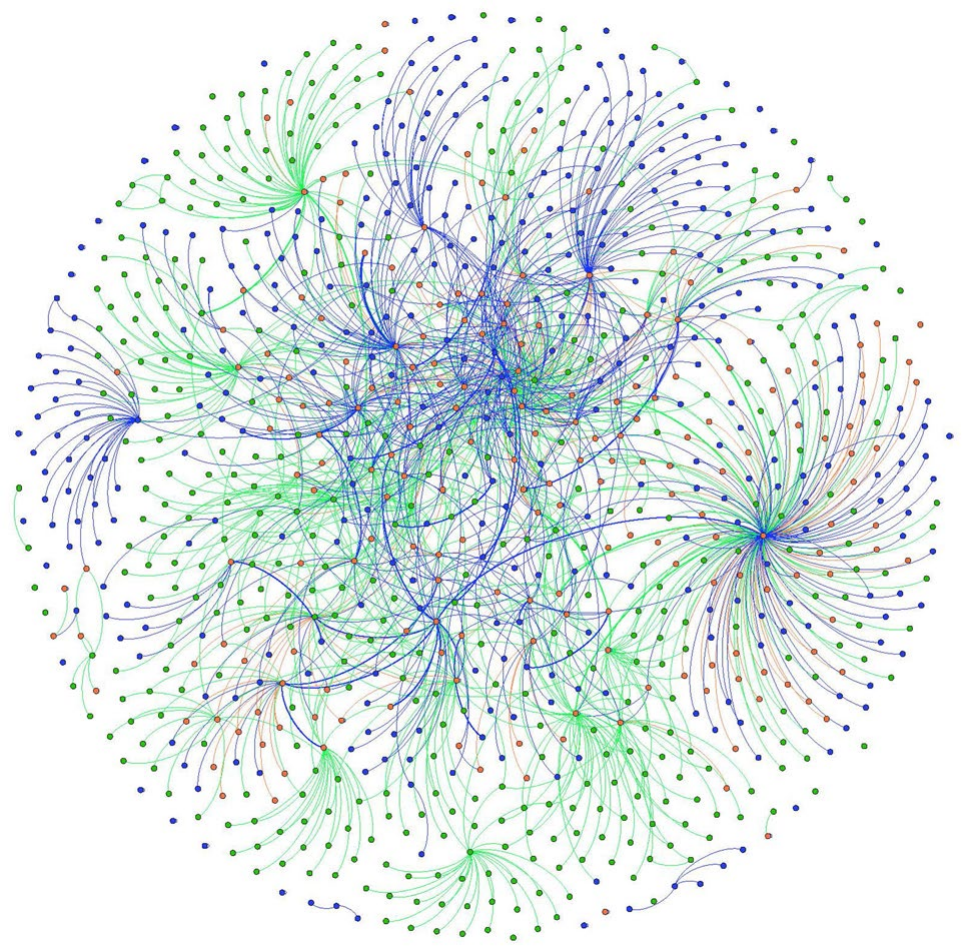
Chester County 2012 and 2018 local food network, Fruchterman Reingold layout. 2012 nodes and edges are in green 2018 nodes and edges are in blue. Nodes and edges that were in both years are in orange. (Color figure online)

**Fig. 2:**
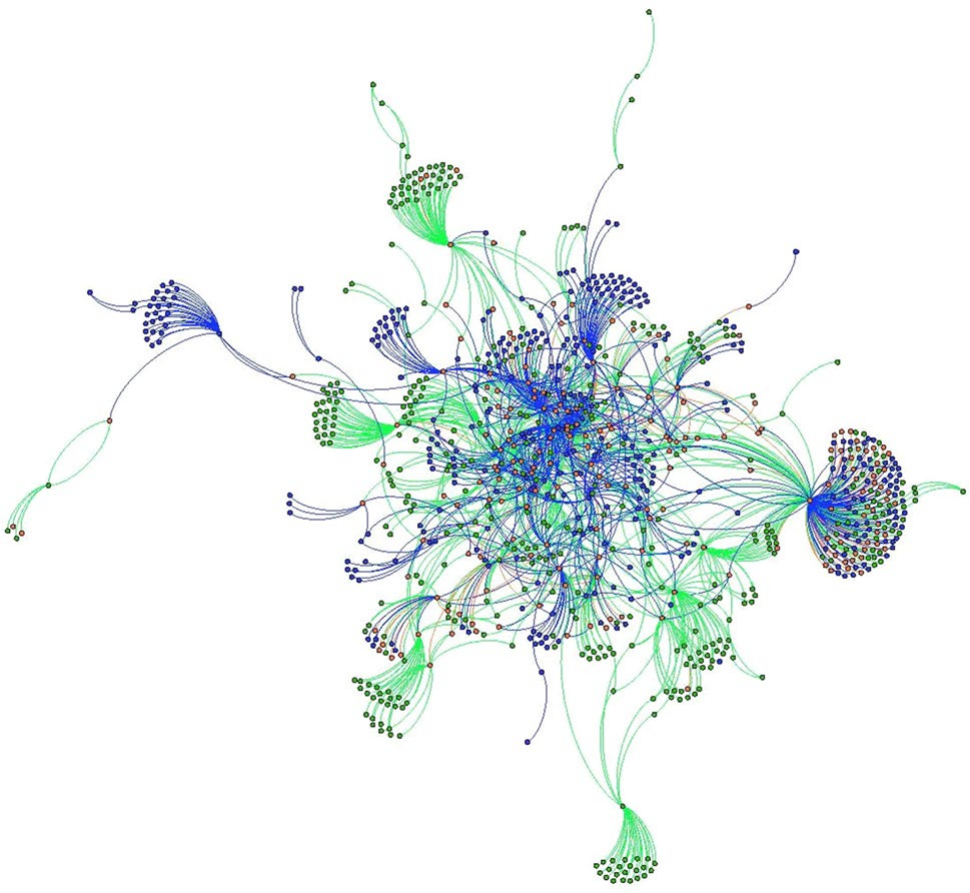
Chester County 2012 and 2018 local food networks, YiFan Hu layout. 2012 nodes and edges are in green 2018 nodes and edges are in blue. Nodes and edges that were in both years are in orange. Image shows only nodes that were connected to the network— isolates have been removed. (Color figure online)

**Fig. 3:**
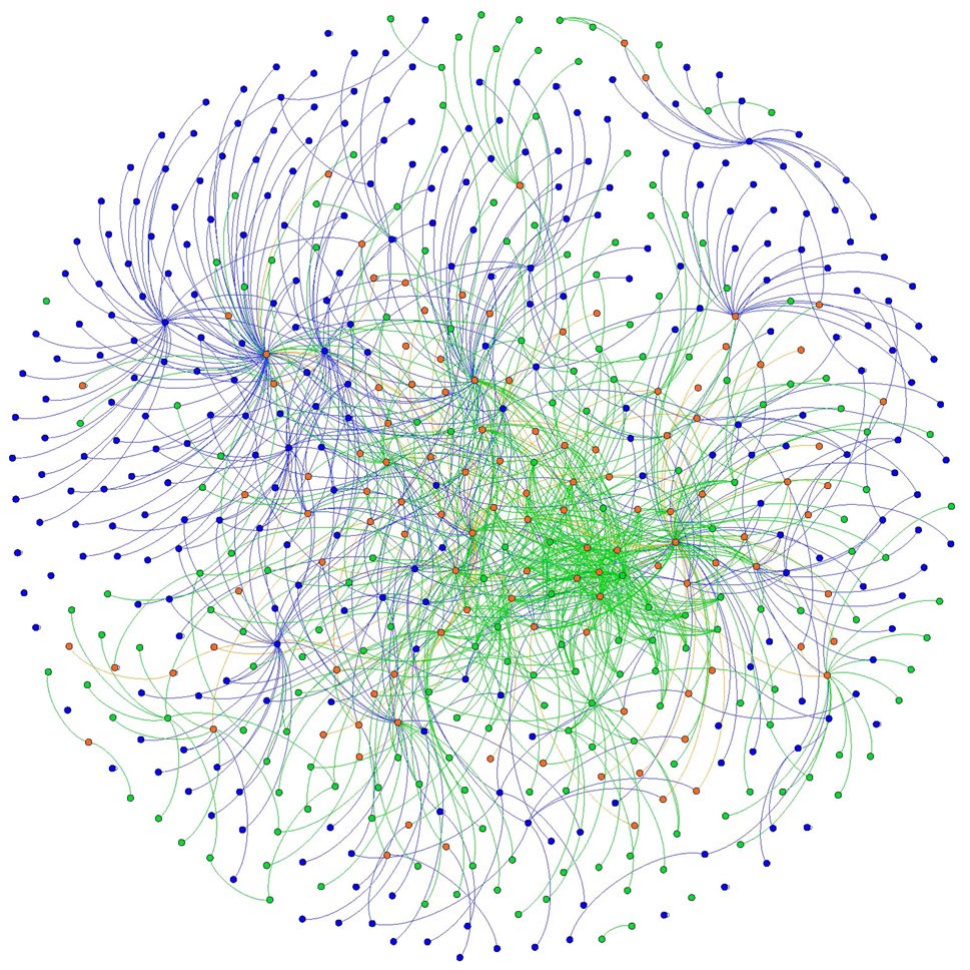
Baltimore County 2012 and 2018 local food network, Fruchterman Reingold layout. 2012 nodes and edges are in green 2018 nodes and edges are in blue. Nodes and edges that were in both years are in orange. (Color figure online)

**Fig. 4:**
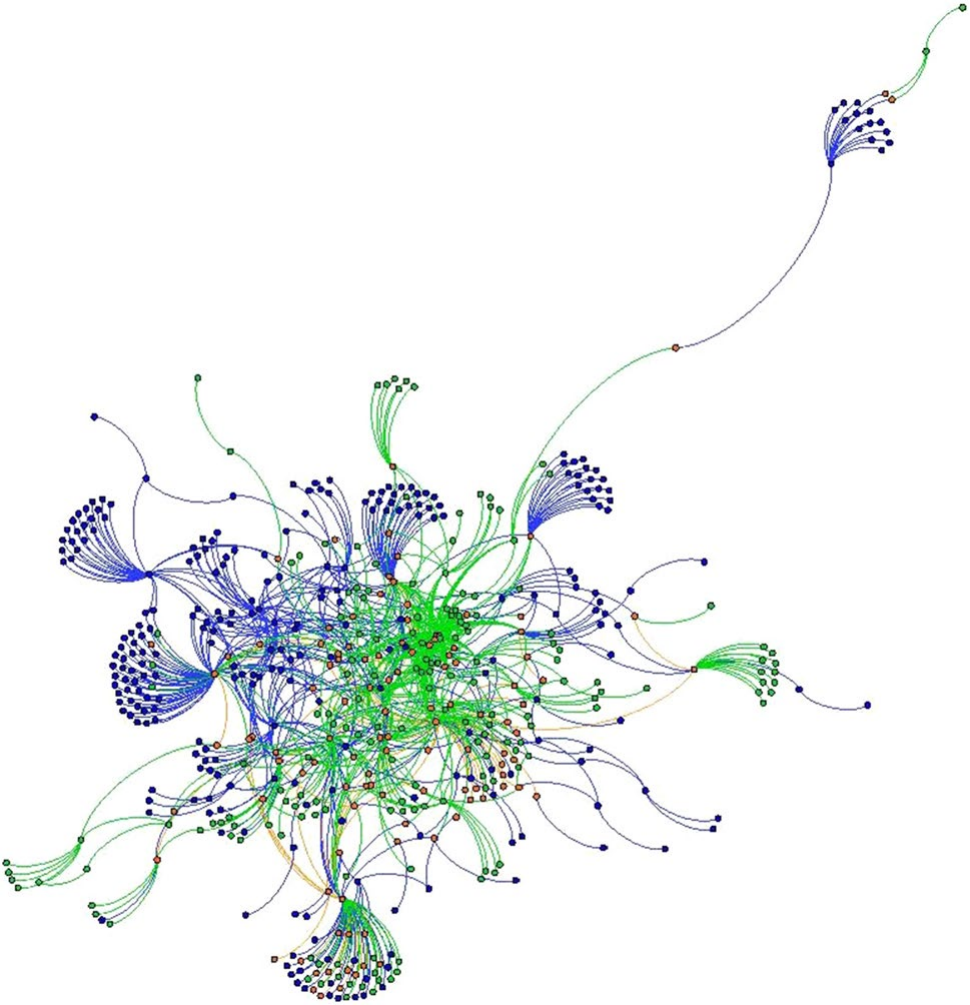
Baltimore County 2012 and 2018 local food networks, YiFan Hu layout. 2012 nodes and edges are in green 2018 nodes and edges are in blue. Nodes and edges that were in both years are in orange. Image shows only nodes that were connected to the network— isolates have been removed. (Color figure online)

To understand if markets are growing outward socially or if new members are incorporated at the heart of the network, we use SNA visualization to show how the web of market ties have changed over time. When visualized socially, with the most connected actors at the center of the network, Chester County’s local food system shows growth and decay concentrated along the network’s outer margins, though growth and death within the network is widespread (Figs. 1 and 2). In contrast, Baltimore County shows significant network decay amongst actors that were central to the network in 2012, with growth occurring on the network’s periphery (Figs. 3 and 4). Broadly, such patterns may be the hallmarks of a larger, more established local food system in Chester County evolving at the margins, with stable central network actors maintaining the core relationships and network architecture. Conversely, Baltimore County appears to be reinventing itself, with high turnover in actors that were once central to the network.

Basic network statistics help reinforce the findings from visualizations, while telling a more nuanced story about the evolution of the local food systems in both counties (Table 3). To quantify how connected the local food system is, we use the *average degree* statistic*,* which indicates the average number of actors to which each node is tied. Chester County had a stable average degree measure between 2012 and 2018, while the average degree of Baltimore County declined substantially from 2.023 to 1.37, meaning that actors within the local food system have fewer average connections in 2018 than they did in 2012. The *clustering coefficient* indicates the degree to which the neighbors of a node are connected. A coefficient of 1 would indicate that all neighbors are connected to each other, while a coefficient of 0 would indicate that none of a node’s connections have mutual ties. While the average clustering coefficient for Chester County remained stable at 0.0023 between 2012 and 2018, the clustering coefficient for Baltimore County dropped from 0.032 to 0.023. In sum, Baltimore’s network became sparser and more porous due to the many confirmed closures, mentioned above, that were central to the network architecture (Figs. 3 and 4). As central actors dropped out of Baltimore County’s local food system (Figs. 3 and 4), newer actors grew at the network’s fringe. However, this growth was not fast enough to reestablish the same level of connectivity across the network.

To understand how information might travel across the network, we use *network diameter*, which indicates the maximum distance between any two nodes within the network. The *network diameter* shrank for both networks, indicating that the overall local food system became more close-knit (Table 3) potentially enabling information to travel across market ties more quickly. Similarly, the *average path length* for both networks also declined. The average path length indicates the average steps needed to get from one actor in the network to another and is often used to gauge how quickly information can travel across a network. Declines in network diameter and average path length indicate the development of a more tightly integrated and consolidated local food system. Had the network split, the path across would have become disconnected or very long. Such splits can occur when social or market networks fraction, but this was not the case in either county. Finally, *graph density* shows the total number of edges within the network relative to the possible number of edges within a network. In other words, if every node within a network were connected to every other node in the network the density value would be 1, while if no nodes were connected to each other the density value would be 0. Both networks saw graph density decline between 2012 and 2018. As both local food systems are maturing, they are consolidating and reducing the redundancy in connections.

### Centrality of actors

The perseverance of actors and ties across both years could be interpreted as strong ties among actors, while new connections and nodes may represent innovation and “weak ties.” Between 2012 and 2018 the actors most central to both networks cultivated new sales and market channel relationships, both with actors that were new to the network and with enduring actors with whom they were not previously connected. This finding indicates innovation among both enduring and new network actors. Collectively, the above statistics demonstrate that the total makeup of the network is in considerable flux.

Additionally, the data indicate that the centrality of actors is changing. *Betweenness centrality* indicates the extent to which a node acts as a bridge between two other nodes. As such, high betweenness centrality can suggest a node’s substantial power within a network, as it may serve as a broker between other actors. In Baltimore County, only one node (Springfield Farm) was ranked in the top ten highest betweenness centrality in both 2012 and 2018. Similarly, within the Chester County dataset, only one node (the Chester County Food Bank) was ranked in the top ten highest betweenness centrality across both years. Previous research has demonstrated the role that these specific actors have played in brokering new partnerships across the food system and influencing land-use policy (Brinkley 2017, 2018). The turnover of other actors central to the network was an unexpected finding, showing deep changes within the local food system as the constellation of people and organizations changed. These changes likely translate to shifts in the sphere of influence of these actors as well.

Scholarly literature has portrayed growing local food systems as creating enduring, embedded ties while also having a high turnover. While these claims appear paradoxical, this research helps show why such assertions may be simultaneously true. The persistence of high-centrality nodes, like the Chester County Food Bank and Springfield farm, and strength of their ties across the local food system may be especially important in an ever-changing network that is dominated by weak ties. Such weak ties foster innovation (Granovetter 1977, 1983) as new forms of market channels and associated socio-political alliances are formed across the local food system.

### Network spatiality

**Fig. 5:**
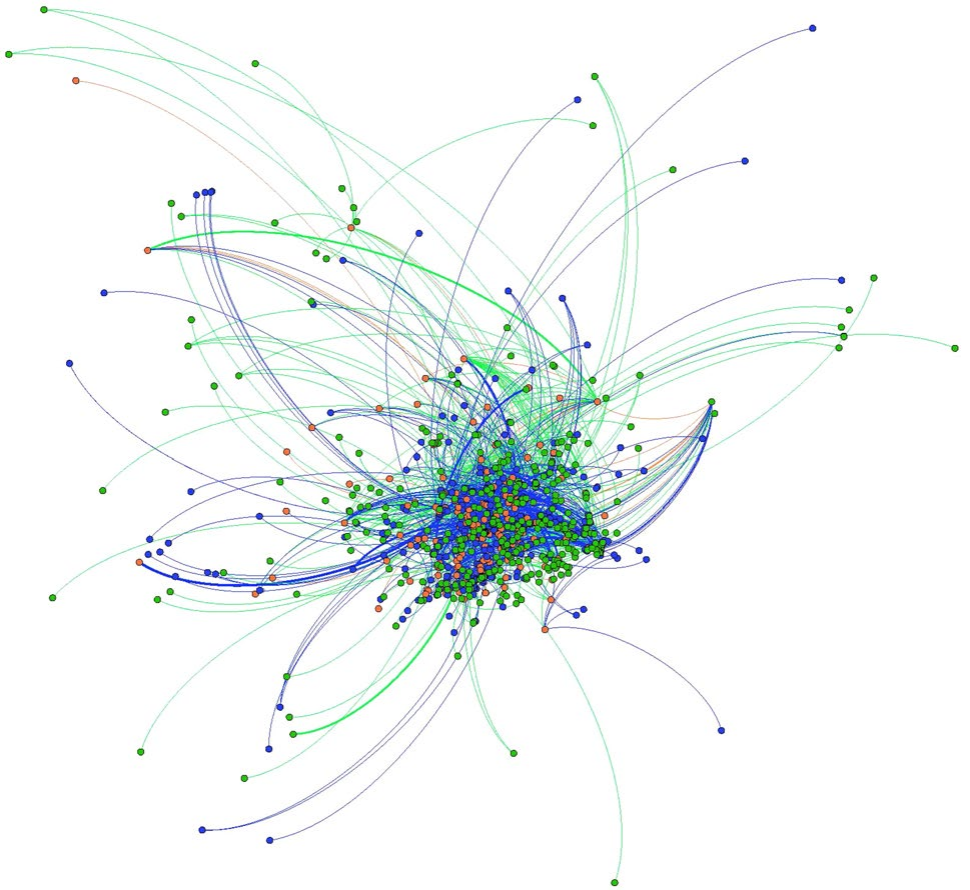
Chester County 2012 and 2018 local food network, Geolayout. 2012 nodes and edges are in green 2018 nodes and edges are in blue. Nodes and edges that were in both years are in orange. Network map shows 98% of network nodes and 95% of edge connections. (Color figure online)

**Fig. 6:**
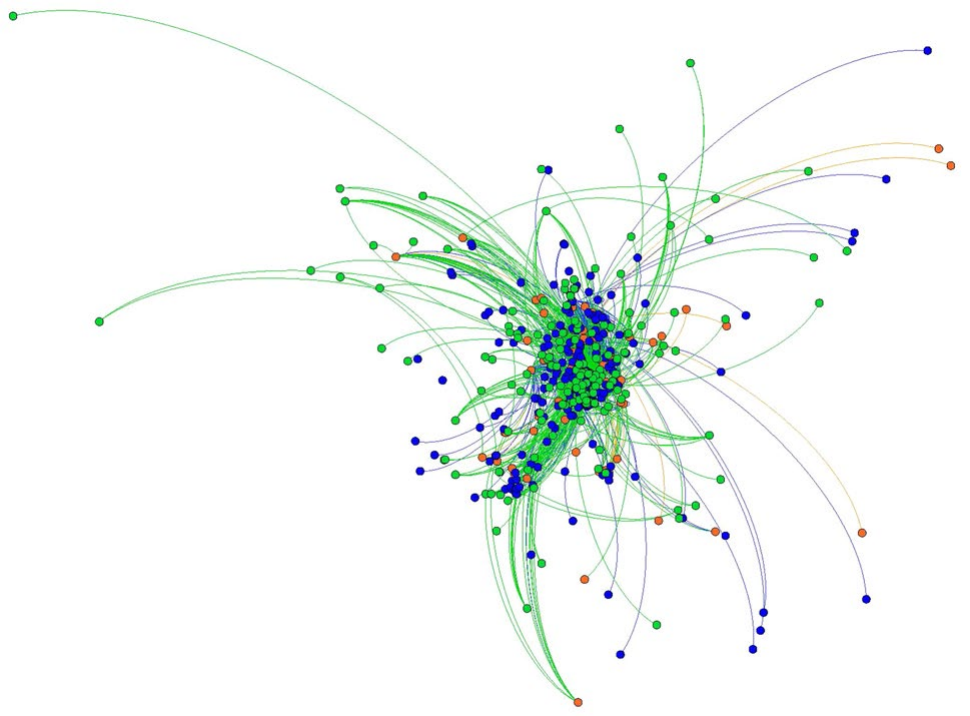
Baltimore County 2012 and 2018 local food network, Geolayout. 2012 nodes and edges are in green 2018 nodes and edges are in blue. Nodes and edges that were in both years are in orange. Network map shows 99% of network nodes and 90% of edge connections. In both geo layout figures, north is up. (Color figure online)

Last, spatial trends related to network change over time help build on earlier research that considers the growth of local food systems as a response to the bow wave of urban development (Hart 1990; Zasada 2011; Brinkley 2012). The Chester County dataset shows growth of the local food network in the northern parts of the county (Fig. 5), and a simultaneous loss of food system actors in the southern portions of the county. Actor loss was clustered close to the City of Philadelphia. In Baltimore County (Fig. 6), network actors that were present across both years of the dataset were engaged in forming new edges and maintaining old connections. Similar to Chester County, actor loss is clustered in the southern portion of Baltimore County, which is closest to the City of Baltimore. Growth within the network is clustered to the north, which corresponds with Baltimore County’s more rural areas.

In both counties, the local food system experienced actor loss closer to urban areas, and new growth further from cities in peri-urban and rural areas. It is important to note that actors are not only farms, but also other nodes, such as farmers’ markets. This finding suggests that there may be spatial boundaries to the ideological objectives of the local food movement. As farms are forced further away from urban areas, the distances to get to urban markets may become too far to traverse. At the same time, suburban growth may also stretch the social distance between urbanites and rural dwellers, placing the many shared objectives of the local food movement further from people’s reach, both physically and mentally.

While the counties have many differences, the similarities across both datasets may point to larger trends regionally or nationally in local food marketing. We show that farms are joining the local food movement. This change is not captured in the USDA agricultural census for either county, though it is noted nationally. The number of farms with DTC sales increased by 5.5% from 2007 to 2012, but with no increase in DTC sales (Low et al. 2015), and then the number of farms with DTC sales declined in 2017 (O'Hara and Benson 2019). Like the USDA agricultural census, we found that the most common way of selling local food was through intermediate markets, and that online marketing appeared to be on the rise. Marketing pathways are rapidly changing. In addition, both networks are consolidating and becoming more tight knit. Such change would indicate that these local food systems are made up of weak ties, enabling rapid innovation, with ever decreasing distances from one side of the network to the other. As a result, news travels faster. The network architecture of these two cases reveals that despite these weak ties both counties have a stable central actor that maintains the core identity of the county through political engagement with land-use policy and planning. These network findings help make sense of seemingly conflicting accounts that local food systems struggle *and* are growing; innovate *and* are historic (Pretty 1990; Vitiello and Brinkley 2014); and last, that they are dominated in numbers by weak ties *and* in central actors with strong bonds.

## Discussion and conclusion

This research challenges common narratives about local food systems. The substantial flux captured across both food systems has not been anticipated in past literature, which often frame local food systems in terms of stable growth, but overlook their simultaneous decay. We found that the local food systems in both northeastern counties reinvented themselves by half and rewired nearly 80% of their connections within 6 years (Table 1). Identifying drivers of growth, stability, and decay are important for generalizing findings further.

While past literature acknowledged that local food systems are multifaceted (Born and Purcell 2006), complex, and adaptive (Nelson and Stroink 2014; Blay-Palmer et al. 2016), the extent and timescale of their evolution generates new questions about how rapidly the social movements they represent shift socio-political focus, and their constituents along with them. There is evidence of these shifts at the national scale. For example, the rise of food justice movements highlights the lack of access to land ownership and markets for farmers of color. As these movements continue to gain momentum, task forces made up of growers and market managers of color are producing policy platforms. Soul Fire Farm in New York and the Northeast Farmers of Color alliance put forth a ‘Food Sovereignty Proposal’ in Soul Fire Farm and Northeast Farmers of Color Alliance 2018, which was acknowledged in Elizabeth Warren’s national presidential campaign (2020). SNA, in combination with qualitative research, could highlight where and how "Buy Black” campaigns (Hinrichs and Allen 2008) or boycotting certain stores changes marketing networks and their embedded power structures. Similarly, SNA in combination with spatial regression analysis can trace if local food is increasingly moving to whiter more affluent block groups and where it interfaces with lower income communities and majority-minority block groups.

Our research suggests that forming a “network of networks” (Levkoe 2014; Blay-Palmer et al. 2016) to scale up the political ambitions of broader food movements may prove especially challenging given the high flux and heterogeneity at the local level, but such an effort could happen rapidly given how local food networks are already reorganizing. To this end, social movement scholars note that the impact of a social movement on political change is understudied (Burstein et al. 1995) and that the outcomes over time must be measured against shifts in network composition, political focus and tactics (Andrews 2001). As this research reveals, the very social architecture of local food systems is shifting. One would expect the political objectives to also change.

The decay of the network, particularly at the heart of the local food system in Baltimore County, prompts further considerations. How much can a “social network” change and still endure? The answer depends partly on how rapidly the network replenishes its ties and actors, and how adept it is at recruiting. Our research suggests that a complete disruption in recruitment into the local food system could see the food system itself cease to exist in a 12-year time frame if it followed a linear pattern. There may be cascading events where closures create ripple effects and network disruption occurs more quickly than expected. Based on the architecture, we suspect a long-tailed distribution of network ties, which would indicate that growth and death is exponential, not linear. Such considerations are important to understanding how local laws restrict the ability of new local food systems to grow, endure, and thrive. For example, cities limit permits for new farmers’ markets (Brinkley 2017), and nations direct agricultural subsidies in a manner often counter to local food systems (Randall 2002; Marsden and Sonnino 2008). Framed another way, with more supportive policies, our research gives clues to how quickly a local food system might blossom. There are ample examples from the organizational literature with regard to how agricultural policies create new marketing networks; allowing, for example, the rapid agricultural transformation in Cuba (Messina 1999). If network growth socially builds outwards from a stable core, as it has in Chester County, non-linear, exponential growth can be expected.

Shifts in network alliances are of particular concern in understanding how communities regulate land-use. Spatial findings help reinforce research that considers the rise of local food as a response to a wave of urbanization (Brinkley 2012). Further, the “eat local” political focus of local food systems, particularly around county-level land-use policies (Brinkley 2018), suggests that as the system rewires, it may reactively form new alliances in anticipation of major planning efforts. Both Chester and Baltimore Counties showed network growth in more rural areas, and network decay closer to the urban centers. These findings lend support to John Hart’s concept of a perimetropolitan bow wave, in which metropolitan areas steadily encroach upon, and eventually engulf, adjacent peri-urban farmland (1991). Even prior to engulfment, encroachment has implications for farming operations—as the bow wave approaches and land values rise, farmers often shift their production and market channels (Zasada 2011). Our findings demonstrate where constituents are turning to local food systems as an antidote. During this study period, the housing market was steadily recovering from the 2008 recession. The shift of local food systems further from urban areas may differ under different housing markets or economic recessions, a topic for future research on just how reactive or protective the local food movement may be for slowing suburbanization. The spatial aspects of network decay also indicate that land-use patterns that keep rural and urban land-uses in close proximity may help foster greater network ties and stability across the network. In turn, such market connections should reinforce rural–urban social relationships that produce mutual understandings and a shared political agenda.

The use of SNA uniquely highlights the disproportionate impacts that a few organizations or individuals can exert on total network stability. The Chester County Food Bank’s role in promoting new farms and markets while connecting them to civic society (Brinkley 2017) undoubtedly contributes to their own stability and centrality to the network, but also to the broader objectives of the local food movement in Chester County to preserve farmland and provide food security. This study was conducted during a time period with relatively low unemployment rates, but economic recession will add pressure for food banks to mobilize food and volunteers, and serve more people. Chester County’s food bank is well-positioned (centrally, even) in mobilizing the local food system to such a daunting task. Other food banks nationally are also interfacing with local food movements (Vitiello et al. 2015). Such findings highlight the ties between local food and food security, and open new avenues of research into how food banks both sustain the local food movement’s transactional markets, and interface with its political objectives.

Broader trends within marketing categories offer further timely generalizations for how to sustain local food systems during times of crises. Many states have banned restaurant dining during the COVID-19 pandemic, and quarantine protocols have placed considerable economic pressure on small businesses. Half of small businesses have enough cash to survive for 27 days without new revenue; restaurants have 16 buffer days on average (Farrell and Wheat 2016). Local food systems with larger percentages of restaurants and which are more dependent on restaurants for network growth, like that of Baltimore County, (Table 1) will likely have larger blows dealt to the local food system than counties that are not as reliant on restaurants. Widespread restaurant closures may have ripple effects across the local food movement, impacting collective action and mobility for a variety of topics ranging from food justice policies to land-use planning. While turnover in the restaurant business is well documented, with the median restaurant lifespan of 4.5 years (Luo and Stark 2014), this research raises questions about the median lifespan of other businesses, such as CSA farms and farmers’ markets, and the impact of market outlet closure on small-scale farms. SNA also demonstrates that the closure of just a few nodes can substantially alter network connectivity, be those restaurants or other node typologies. Such findings help reinforce the notion that collective action in the food movement is dependent on many forms of food sales and donations.

Using a longitudinal SNA approach to compare the evolution of two local food systems opens the doors for a number of future studies. This data raises questions about what methods of direct marketing are most vulnerable to disappearance and change. Chester County, Pennsylvania saw a significant reduction in the number of CSA connections in the network. Are CSAs used as stepping stones towards other forms of direct and indirect sales relationships? Online and platform-based marketing introduce new questions about embeddedness characteristics as the local food system moves from a face-to-face interaction to a virtual “know your farmer” experience. Will these new forms of embeddedness flavor the endurance or loyalty of network actors, and differently influence civic engagement? The collection of qualitative data through interviews and surveys could add additional detail to these findings. Indeed, this research does not cover changes in consumer ties to markets, which would presumably influence staying power. Consumer ties likely have important impacts on overall network architecture, as well as associated local policy objectives and outcomes.

Future studies may replicate findings and move the literature toward a typology of local food systems. Some, like Chester County, may be relatively stable, with the addition of new network members and connections on the periphery of the network (Figs. 1 and 2). Others, like Baltimore, could be reinventing themselves at their very core (Figs. 3 and 4). Understanding how such changes in network architecture broadly correlate to shifts in policy objectives will yield new insights into how a network of local food networks could be scaled up globally, currently a theoretical concept for broad social change (Blay-Palmer et al. 2016).

## Appendix

See Tables 4, 5 and 6.

**Table 4:** Agricultural production typology codes

Production type	Description
Dairy	Cow/goat products (milk, cheese, etc.)
Meat	Any form of terrestrial animal meat
Seafood	Any form of seafood
Honey	Raw honey
Nuts	Nuts
Produce	Berries, vegetables, fruit, herbs
Eggs	Any type of eggs (chicken, duck, etc.)
Mushrooms	Edible fungi
Plants	Edible plant starts
Grain	Grain that is explicitly turned into flour

**Table 5:** Code guide for node typologies (types of actors)

Node Type	Description
Farmers’ market	Self-identified farmers’ market
Grocery Store/wholesale	Includes national chains, co-ops, and small markets (but not farm stores)
Distributor/food hub	A third party that serves as an intermediary between producer and consumer; examples: food hub, home delivery service of multiple producer's goods
Catering company	Company that offers on- or off-site catering (may also be associated with a restaurant)
School	PK-12 or college/university. Also includes dining service operators that work within schools
Health/medical facility	Medical hospitals, wellness institutes or any other medical treatment centers (i.e. chiropractor), yoga studios, gym
Farm stand	Farm sells their products at an on-site farm stand or store
Value added producer	A company that buys produce/product directly from the farm to produce a value added product (such as a hot sauce or jam company that does not operate a restaurant)
Restaurant	Includes brick and mortar locations, coffee shops, bakeries, food trucks, and farmers’ market vendors who turn farm goods into value added products that they sell at a farmers’ market
Farm	A farm that produces meat, produce, eggs, mushrooms, etc. (see production types below)
Liquor store	A location that primarily sells liquor/wine (a wine bar would not be located under this category)
Office	Workplace (may function as CSA pickup location, for example)
Church	any place of religious/spiritual worship
Box scheme	Produce or raw agricultural product delivery services. Distinct from a single farm using an online sales platform to sell their own products
Winery	Self-identified winery
Other	Miscellaneous locations
Private residence	Self-identified private residence
Community center	Community center, such as a YMCA or a senior center/senior living center
Food bank	Self-identified food bank
Cannabis store	Self-identified cannabis store
Garden store	A retail location that primarily sells garden items (decorative plants, soil)
You-Pick	Farms with a "you pick" option where customers can come directly to the farm to pick their own produce
Mobile farmers’ market	Farmers’ market "truck" that brings fresh produce to communities
Cidery	Self-identified cidery
Hobby gardener	Someone who grows in their backyard but sells some products
Community garden	In Chester a lot of the community gardens are donating to the food bank
Butcher	Butcher shop

**Table 6:** Code guide for edge typologies (types of relationships)

Code	Description
Farmers’ market	Self-identified farmers’ market
Grocery Store/wholesale	Includes national chains, co-ops, and small markets (but not farm stores)
Distributor/food hub	A third party that serves as an intermediary between producer and consumer; examples: food hub, home delivery service of multiple producer's goods
Catering company	Company that offers on- or off-site catering (may also be associated with a restaurant)
Farm to farm	One farm or producer selling directly to another farm or producer
School	PK-12 or college/university. Also includes dining service operators that work within schools
Hospital	Medical hospitals
CSA pickup (customer to farm)	Customer goes to the farm to pick up their CSA share
CSA pickup (farm to location)	A location other than the farm at which a customer can pick up their CSA share
Farm visits	Farm offers farm visits for schools/education
Farm stand	Farm sells their products at an on-site farm stand or store
Value added producer	A company that buys produce/product directly from the farm to produce a value added product (such as a hot sauce or jam company that does not operate a restaurant)
Restaurant	Includes brick and mortar locations, coffee shops, bakeries, food trucks, and farmers’ market vendors who turn farm goods into value added products that they sell at a farmers’ market
Donation	Donation sites, such as food banks or churches
Box scheme	Produce delivery services
Online sales	Online sales direct from the farm
You-pick	Farms with a "you pick" option where customers can come directly to the farm to pick their own produce
Mobile farmers’ market	Farmers’ market "truck" that brings fresh produce to communities
Butcher to farm	A butcher shop that sells their meat at farm stands
Donation (raised beds)	Donations from raised bed gardens to the Chester county food bank (particular to Chester)
Fresh2you	A specific program of the Chester county food bank for distributing fresh food in the county
Farm to winery	Farms that sell fresh product to wineries (for the restaurant of the winery)

## Abbreviations

AFN: Alternative food network
CSA: Community Supported Agriculture
DTC: Direct-to-consumer
SNA: Social network analysis
USDA: United States Department of Agriculture
